# Using a 29-mRNA Host Response Classifier To Detect Bacterial Coinfections and Predict Outcomes in COVID-19 Patients Presenting to the Emergency Department

**DOI:** 10.1128/spectrum.02305-22

**Published:** 2022-10-17

**Authors:** Nikhil Ram-Mohan, Angela J. Rogers, Catherine A. Blish, Kari C. Nadeau, Elizabeth J. Zudock, David Kim, James V. Quinn, Lixian Sun, Oliver Liesenfeld, The Stanford COVID-19 Biobank Study Group, Samuel Yang

**Affiliations:** a Department of Emergency Medicine, Stanford University School of Medicine, Stanford, California, USA; b Department of Medicine—Pulmonary, Allergy & Critical Care Medicine, Stanford University School of Medicine, Stanford, California, USA; c Department of Medicine/Infectious Diseases, Stanford University School of Medicine, Stanford, California, USA; d Inflammatix, Inc., Burlingame, California, USA; University of Warwick

**Keywords:** diagnosis, COVID-19, bacterial superinfection, coinfection, prognosis, mortality prediction, host response classifier, emergency department

## Abstract

Clinicians in the emergency department (ED) face challenges in concurrently assessing patients with suspected COVID-19 infection, detecting bacterial coinfection, and determining illness severity since current practices require separate workflows. Here, we explore the accuracy of the IMX-BVN-3/IMX-SEV-3 29 mRNA host response classifiers in simultaneously detecting severe acute respiratory syndrome coronavirus 2 (SARS-CoV-2) infection and bacterial coinfections and predicting clinical severity of COVID-19. A total of 161 patients with PCR-confirmed COVID-19 (52.2% female; median age, 50.0 years; 51% hospitalized; 5.6% deaths) were enrolled at the Stanford Hospital ED. RNA was extracted (2.5 mL whole blood in PAXgene blood RNA), and 29 host mRNAs in response to the infection were quantified using Nanostring nCounter. The IMX-BVN-3 classifier identified SARS-CoV-2 infection in 151 patients with a sensitivity of 93.8%. Six of 10 patients undetected by the classifier had positive COVID tests more than 9 days prior to enrollment, and the remaining patients oscillated between positive and negative results in subsequent tests. The classifier also predicted that 6 (3.7%) patients had a bacterial coinfection. Clinical adjudication confirmed that 5/6 (83.3%) of the patients had bacterial infections, i.e., Clostridioides difficile colitis (*n* = 1), urinary tract infection (*n* = 1), and clinically diagnosed bacterial infections (*n* = 3), for a specificity of 99.4%. Two of 101 (2.8%) patients in the IMX-SEV-3 “Low” severity classification and 7/60 (11.7%) in the “Moderate” severity classification died within 30 days of enrollment. IMX-BVN-3/IMX-SEV-3 classifiers accurately identified patients with COVID-19 and bacterial coinfections and predicted patients’ risk of death. A point-of-care version of these classifiers, under development, could improve ED patient management, including more accurate treatment decisions and optimized resource utilization.

**IMPORTANCE** We assay the utility of the single-test IMX-BVN-3/IMX-SEV-3 classifiers that require just 2.5 mL of patient blood in concurrently detecting viral and bacterial infections as well as predicting the severity and 30-day outcome from the infection. A point-of-care device, in development, will circumvent the need for blood culturing and drastically reduce the time needed to detect an infection. This will negate the need for empirical use of broad-spectrum antibiotics and allow for antibiotic use stewardship. Additionally, accurate classification of the severity of infection and the prediction of 30-day severe outcomes will allow for appropriate allocation of hospital resources.

## INTRODUCTION

Clinicians in the emergency department (ED) face major challenges in accurately assessing patients with suspected infections, including severe acute respiratory syndrome coronavirus 2 (SARS-CoV-2) and bacterial coinfections as well as predicting clinical outcomes. Bacterial coinfections (at presentation) and superinfections (after presentation) (1, 2) often cause worse outcomes than the primary viral infection (3); this phenomenon was prevalent in the H1N1 influenza pandemic (4), with 20% to 30% bacterial coinfections in patients with severe influenza (5, 6). However, current evidence for COVID-19 portrays a different scenario. Recent studies have shown rates of bacterial coinfection in COVID-19 of between 3.2% and 5.5% (1, 7–9), with rates of secondary or superinfection in hospitalized patients increasing to 6.1% to 15% (1, 7, 10, 11). Despite the relatively low prevalence of bacterial coinfections in COVID-19, empirical antibiotics for community- or hospital-acquired bacterial pneumonia or bacteremia are often prescribed in severely ill patients due to the inability to accurately or rapidly detect bacterial coinfection at presentation (1, 12, 13).

Existing diagnostic tests have major limitations. “Gold standard” bacterial cultures often take days to obtain a result, are limited by the ability of the organism to grow in the culture medium, and require a large sample volume when testing complex patient samples like blood (14, 15). In addition, false negatives can result from insufficient culture duration or antimicrobial treatment prior to sample collection (16). False-negative culture results can have devastating consequences for patients. Alternate testing methods involve PCR-based targeted amplification of bacterial nucleic acids directly from the patient’s blood sample. These are not routinely used in the acute setting, are limited by turnaround time and the panel of targets they can detect, and are influenced by the inherent issues of PCR—lack of sensitivity in detecting low bacterial loads, sensitivity to protocols and threshold decisions adopted, and the presence of inhibitory molecules in complex samples such as blood (17).

There is therefore an unmet medical need to identify viral and bacterial infection using rapid point-of-care tests in the ED to determine presence and severity of infection and inform the use of antimicrobials. In the absence of such diagnostics, clinical decision-making needs to balance antimicrobial stewardship with delivery of appropriate empirical care, including escalation of therapy in patients with suspected bacterial coinfections and/or suspected sepsis, to predict severity for level-of-care decisions, and optimal use of health care resources.

The machine-learning supported host response mRNA classifier IMX-BVN-2 has recently been described to accurately identify systemic as well as localized bacterial infections and also viral infections other than COVID-19 (18). A separate classifier, IMX-SEV-2, has been developed to predict illness severity (19). The identity and biological functions of the 29 host mRNAs have recently been published (20), and the classifiers have been further updated (IMX-BVN-3 and IMX-SEV-3) based on additional clinical study data.

The aim of this study was to investigate the accuracy of IMX-BVN-3 and IMX-SEV-3 classifiers to detect SARS-CoV-2 infection, detect bacterial coinfections, and predict the severity of illness in patients with confirmed COVID-19.

## RESULTS

### Patient characteristics.

A total of 161 patients were enrolled from April 2020 to February 2021, with a median age of 50 years (interquartile range [IQR], 35 to 64). A total of 84/161 (52.2%) were women. A total of 158/161 (98.1%) were symptomatic on presentation, with a median of 6 symptoms (IQR, 4 to 8). Medical history, comorbidities, and symptoms at presentation are shown in Table 1.

**TABLE 1:** Patient medical history and symptoms at presentation

Parameter^*a*^	% (no.) of patients (*n* = 161)
Medical history	
Lung disease	14.9 (24)
Cancer	6.8 (11)
Diabetes	29.8 (48)
Immunosuppression	9.3 (15)
Heart disease	9.9 (16)
Hypertension	39.1 (63)
ACE/ARB use	21.7 (35)
Stroke	3.7 (6)
Dementia	2.5 (4)
DVT/PE	5.6 (9)
Chronic kidney disease	9.3 (15)
Smoking	21.1 (34)
Symptom(s) at presentation	
Fever	57.1 (92)
Chills	34.2 (55)
Cough	68.9 (111)
Sore throat	21.1 (34)
Congestion	9.9 (16)
Shortness of breath	62.1 (100)
Chest pain	34.8 (56)
Myalgia	41.6 (67)
Nausea/vomiting/diarrhea	56.5 (91)
Loss of taste	39.8 (64)
Loss of smell	36.6 (59)
Confusion	0 (0)
Headache	39.8 (64)

a ACE/ARB, angiotensin-converting enzyme inhibitors/angiotensin II receptor blockers; DVT/PE, deep vein thrombosis/pulmonary embolism.

### Accuracy in predicting COVID-19 infection using host response markers.

A total of 151/161 (93.8%) of patients positive for COVID-19 by RT-PCR were accurately classified as “Possible” or “Very Likely” viral infection by IMX-BVN-3, corresponding to an overall sensitivity of 93.8% (86.3% and 7.5% for the Very Likely and Possible viral bands, respectively; Table 2). As all patients were confirmed SARS-CoV-2 positive, we did not calculate specificity of the classifier.

**TABLE 2:** Breakdown of patients into viral likelihood interpretation bands using IMX-BVN-3^*a*^

IMX-BVN-3 viral interpretation band	Frequency	Sensitivity for COVID-19 (%)
Very Likely	139	86.3
Possible	12	7.5
Unlikely	5	
Very Unlikely	5	

a Specificity was not calculated as all patients were confirmed SARS-CoV-2 positive by PCR.

We further investigated the causes of 10 potentially “false negative” results in BVN-3: six of the 10 patients had first tested positive for SARS-CoV-2 at least 9 days before presentation to the ED, while the remaining four had SARS-CoV-2 PCR test results that were initially positive but oscillated between positive and negative when retested. Of interest, 3 of the 10 patients were predicted to have a bacterial coinfection as indicated by the BVN-3 classifier’s bacterial score and 2/3 were clinically adjudicated to have a bacterial infection by expert chart review (described below).

**FIG 1:**
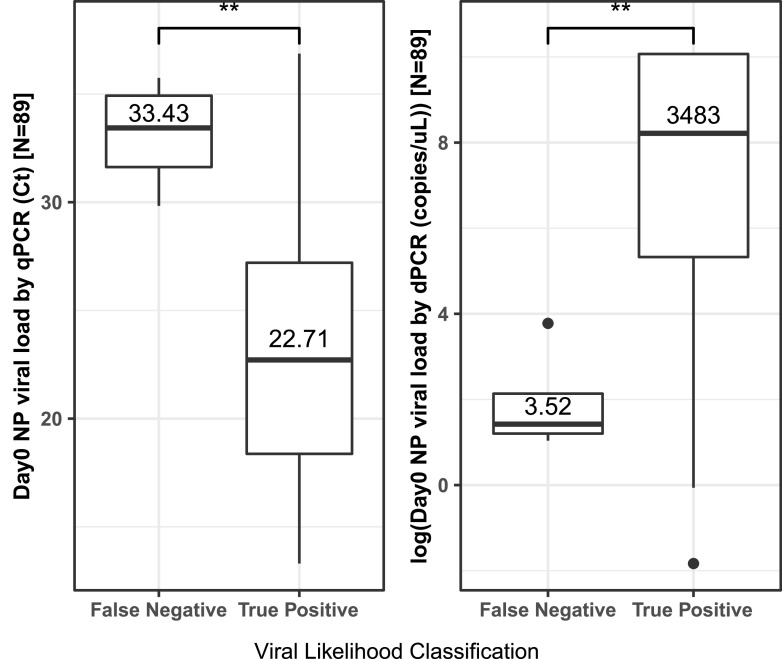
Difference in the nasopharyngeal SARS-CoV-2 load between “false negative” (Unlikely or Very Unlikely viral BVN-3 scores) and “true positive” (Possible or Very Likely BVN-3 scores) for 89 patients. Shown are qPCR-measured viral loads in cycle threshold (*C_T_*) (left) and dPCR-measured absolute viral loads in copies per microliter (right). **, *P* < 0.001.

The viral likelihood score was inversely correlated with the PCR cycle threshold (Ct) value from nasopharyngeal samples collected on admission (Spearman rank correlation, −0.63; *P* < 0.001) and correlated with the absolute viral load (copies per microliter) as determined by digital PCR (dPCR) (Pearson correlation, 0.52; *P* < 0.001) (Fig. 1). Patients with Very Likely or Possible positive BVN-3 viral scores indicating viral infection (“true positives”) had a median viral load of 3,483 copies/μL in the nasopharyngeal sample (IQR, 155 to 23,539) compared to 3.52 copies/μL (IQR, 2.82 to 4.9) in the “Unlikely” and “Very Unlikely” BVN-3 (“false negative”) patients (*P* = 0.009).

### Detection of bacterial coinfections using host response markers.

The IMX-BVN-3 bacterial score classified 6/161 (3.7%) of patients into the Possible bacterial interpretation band, suggesting a bacterial coinfection, and 155/161 (96.3%) were classified as Unlikely or Very Unlikely bacterial infections (Table 3 and see Fig. S1 in the supplemental material). Chart review and clinical adjudication confirmed that 5/6 (positive predictive value, 83.3%) of the Possible bacterial patients did indeed have coinfections, translating into a specificity (ruling in) of 99.4% for identification of bacterial infection in the entire cohort: one patient had Clostridioides difficile colitis, one had rectal adenocarcinoma with gastrointestinal perforation and abdominopelvic abscess, and three had clinically diagnosed coinfections without positive microbiology findings (including blood culture) (Table S1). We did not detect evidence for bacterial infections in 52 of the 58 patients with negative blood culture results, translating into a sensitivity and negative predictive value of 100% for ruling out bacterial infection in the subgroup of patients where microbiology data were available. The bacterial scores correlated with the levels of C-reactive protein (CRP) (Pearson correlation, 0.58; *P* < 0.001), procalcitonin (Pearson correlation, 0.4; *P* = 0.003), and lactate dehydrogenase (LDH) (Pearson correlation, 0.42; *P* = 0.003).

**TABLE 3:** Breakdown of patients into bacterial likelihood interpretation bands using IMX-BVN-3^*a*^

IMX-BVN-3 bacterial interpretation band	No. of patients with:		% in band	Sensitivity (%)	Specificity (%)	Likelihood ratio
	Confirmed bacterial infection	No bacterial infection				
Very Likely	0	0	0	ND^*b*^	ND	ND
Possible	5	1	3.7	ND	99.4	156
Unlikely	0	59	36.6	100	ND	0
Very Unlikely	0	96	59.6	100	ND	0

a Complete data to calculate sensitivity were available in only 58 patients.

b ND, not determined.

### Disease severity based on host response markers and association with clinical outcomes.

The IMX-SEV-3 test classified 101/161 (62.7%) patients in the Low severity category and 60/161 (37.3%) in the Moderate severity category. No patients were categorized in the High severity category. The calculated severity score was correlated with the absolute viral load in plasma (Pearson correlation, 0.49; *P* = 0.002) and the above bacterial score (Pearson correlation, 0.45; *P* < 0.001). Interestingly, 6/6 (100%) patients classified in the Likely bacterial coinfection category were classified to have Moderate severity. The IMX-SEV-3 severity score also correlated with the modified WHO severity score at enrollment for these patients (Pearson correlation, 0.43; *P* < 0.001).

**FIG 2:**
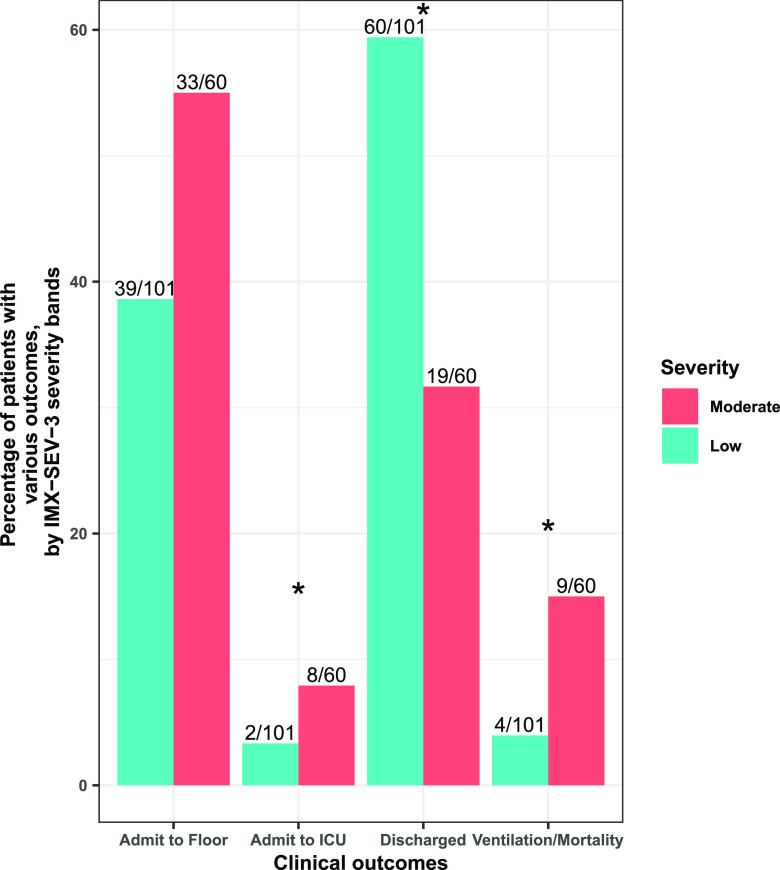
Proportions of patients with different clinical outcomes, including the disposition from the ED as well as the need for ventilation or 30-day mortality, by the severity likelihood predicted by the IMX-SEV-3 classifier. Overall, more patients in the Low category were discharged (difference in proportions, 27.7%; 95% CI, 11.2% to 44.2%) and more patients in the Moderate category were admitted to the ICU or required ventilation/succumbed to the infection, with differences in proportions of 11.4% (95% CI, 1% to 21.7%) and 11.1% (95% CI, 0.09% to 22.2%), respectively. *, *P* < 0.05.

In total, 79/161 (49.1%) patients were discharged, 72/161 (44.7%) patients were admitted to the floor, 10/161 (6.2%) were admitted to the intensive care unit (ICU), 7/161 (4.3%) required mechanical ventilation (Table 4), and 9/161 (5.6%) died. As expected, 59.4% patients in the Low severity category were discharged from the ED compared to only 31.7% in the Moderate category (difference, 27.7%; 95% confidence interval [CI], 11.2% to 44.2%) (Fig. 2). Interestingly, more patients in the Moderate category were admitted to the ICU (difference, 11.4%; 95% CI, 1% to 21.7%). Median IMX-SEV-3 severity scores in patients admitted to the ICU were 14.5 (IQR, 13 to 18.25), in those admitted to the floor was 10 (IQR, 8 to 13), and in those discharged it was 8 (IQR, 7 to 10). The Wilcoxon rank sum test for each pairwise comparison was significant (adjusted *P* < 0.05). When grouping the need for mechanical ventilation and/or mortality as a severe outcome, 13/161 (8.1%) had such a severe outcome from the COVID-19 infection. A greater proportion of patients in the Moderate category had such a severe outcome than those in the Low category (15% versus 3.9%; difference, 11.1%; 95% CI, 0.09% to 22.2%), and the patients in the Moderate category had a higher median IMX-SEV-3 severity score (12; IQR, 10 to 14) than those in the Low category (9; IQR, 7 to 11.15) (Wilcoxon rank sum test, *P* = 0.007).

**TABLE 4:** Breakdown of patients into severity interpretation bands using IMX-SEV-3

IMX-SEV-3 interpretation band	% of patients in band	No. of patients					
		Discharged	Admitted to:		Mechanical ventilation	30-day mortality	Ventilation and/or 30-day mortality
			Floor	ICU			
High	0	0	0	0	0	0	0
Moderate	37.3	19	33	8	4	7	9
Low	62.7	60	39	2	3	2	4

## DISCUSSION

As of January 2022, SARS-CoV-2 has infected more than 340 million people globally and resulted in ~5.5 million deaths (21). Bacterial coinfections/superinfections are known to occur in patients infected with SARS-CoV-2 at various rates of prevalence conditional on the severity of the viral infection (1, 7–13, 22, 23). Successful detection of the virus requires a high-fidelity PCR test targeting the viral RNA, and to date, the detection of coinfecting pathogens has depended on either bacterial culture or detection of target nucleic acids in patient samples using PCR. Here, we present, to the best of our knowledge, the first host response-based simultaneous detection of viral (SARS-CoV-2) infection, coinfection with bacterial pathogens, as well as the stratification of disease severity using the IMX-BVN-3 and IMX-SEV-3 classifiers.

The IMX-BVN-3 classifier detected COVID-19 infection with 93.8% sensitivity. This is the first report of the successful detection of SARS-CoV-2 infection using the IMX-BVN-3 host response signature, which was previously validated in other viral infections (18, 24), and the imputed false-negative rate of the classifier is lower than that of the currently accepted quantitative PCR (qPCR) assays for SARS-CoV-2. A recent systematic review and meta-analysis of 32 studies comprised of 18,000 patients revealed heterologous false-negative rates in qPCR ranging from 2% (25) to 58% (26) with an overall summary estimate of 12% (27). Of interest, we observed several specific circumstances in the few patients that showed “false-negative” results in IMX-BVN-3: first, the time lag between the initial positive SARS-CoV-2 test result and the presentation to the ED in these patients likely indicates clearing of the virus and waning of the associated viral immune response with subsequent negative results in the classifier; second, low viral loads (<5 copies/μL) also contributed to “false-negative” results in the classifier in a few patients. Finally, two patients with “false-negative” classifier results were also found to have bacterial coinfections. As the generation of viral and bacterial scores in the IMX-BVN-3 classifier is interdependent, bacterial scores may have impacted the viral scores and contributed to “false-negative” results in addition to the factors mentioned above.

Importantly, the IMX-BVN-3 classifier predicted bacterial coinfection within 48 h in 6/161 patients with a specificity of 99.4%. Five of six were clinically adjudicated to be bacterially infected. We calculated a prevalence of 8.6% (5/58) for bacterial coinfections in a subset of patients with blood cultures available as part of clinical care; this prevalence is similar to the prevalence reported recently for patients with low or moderate SARS-CoV-2 infection (1, 7–9). Importantly, the identification of bacterial coinfections was achieved from the same 2.5-mL blood sample that provided the viral result in IMX-BVN-3. The high accuracy of the IMX-BVN-3 classifier could thus be used along with the viral result to decide on antimicrobial initiation in the ED, overcoming a major challenge in managing patients suspected with acute infections.

The IMX-SEV-3 classifier categorized patients into Low and Moderate severity categories in our cohort. This host-response-dependent classifier predicted severity scores that correlated with a modified WHO score that was designed to describe the need for supplemental oxygen (28). With a significant difference in the median severity scores of patients admitted to the ICU, admitted to the floor, and those who were discharged, as well as the observed increased proportions of patients in the Moderate severity interpretation band admitted to the ICU and having a severe outcome, the severity score could facilitate the level-of-care decision for ED patients. However, as the study was not powered to assess the accuracy of the severity readout of the IMX-SEV-3 classifier, we only report the nominal results here. Additional studies—including a current large registrational trial conducted for clearance by regulatory agencies in the United States and Europe—will report the accuracy of the severity readout in larger COVID-19 and other cohorts.

Limitations of our study include the fact that this study was conducted at a single center and used biobanked blood samples obtained from a limited cohort of 161 patients. As only PCR-confirmed COVID-19-positive patients were enrolled, we could not determine the IMX-BVN-3 classifier’s specificity. We were also unable to clinically adjudicate the entire patient cohort for bacterial infections and thus calculated sensitivity for a subset of patients only. Finally, since bacterial coinfections or superinfections are defined based on when the patient presents to the ED (1, 2) and not when in the course of the infection the patient presents, we were unable to determine the timeline of the infection to distinguish between the two. Additionally, the host response-based classifier detects any bacterial infection and, hence, does not allow differentiation between coinfections and superinfections.

### Conclusions.

In conclusion, once the IMX-BVN-3 and SEV-3 classifiers are introduced as a rapid point-of-care host RNA detection platform with a turnaround time of less than 30 min (currently in development), results at the point of care could guide decisions about starting or withholding antibiotics, allowing escalation of therapy or antimicrobial stewardship but also the initiation of contact precaution measures and/or viral therapy and choosing the appropriate level of care for SARS-CoV-2-positive patients.

## MATERIALS AND METHODS

### Patient enrollment and specimen collection.

One hundred sixty-one patients with PCR-confirmed COVID-19 infection at presentation were enrolled at the Emergency Department (ED) of Stanford University Hospital, USA. A 2.5-mL whole-blood sample was collected in PAXgene Blood RNA tubes (PreAnalytiX, Hombrechtikon, Switzerland) within 12 h of presenting to the ED and frozen following the instructions of the manufacturer.

Clinical data collected, in the form of a structured questionnaire, included presence of symptoms, past medical history, medications, hospital length of stay (hours and days), CRP, procalcitonin, lactate dehydrogenase (LDH), and ferritin levels and neutrophil, lymphocyte, monocyte, eosinophil, and basophil counts. In addition, we determined the patient’s clinical outcomes in the form of disposition from the emergency department, need for mechanical ventilation, and death.

### PAXgene sample processing.

PAXgene blood RNA tubes were shipped to Inflammatix, Inc. (Burlingame, CA), under a sponsored research agreement where RNA was extracted using a protocol previously described (29), and 29 host mRNAs were quantified using the nCounter FLEX instrument (Nanostring, Seattle, WA).

### IMX-BVN-3 and IMX-SEV-3 classifiers.

Quantification results for the 29 host mRNAs were analyzed using the BVN-3 and SEV-3 host response classifiers. The classifiers generate numerical scores for the likelihood of bacterial infection and the likelihood of viral infection that each fall into 4 diagnostic bands (Very Unlikely, Unlikely, Possible, and Very Likely bacterial and/or viral infection) and a score for the condition’s severity that falls into three prognostic interpretation bands (Low, Moderate, and High severity).

### SARS-CoV-2 RNA quantification.

Plasma and nasopharyngeal viral RNA levels in cycle threshold (*C_T_*) and absolute copies per microliter were determined for 89/161 COVID-19-positive patients coenrolled in our previous study (28) to correlate viral load with the likelihood scores. Briefly, RNA was extracted from 140 μL of samples using the QIAamp viral RNA minikit (Qiagen, Germany) and quantified using the |Q| triplex assay with the qPCR platform QuantStudio 5 (Applied Biosystems by Thermo Fisher Scientific) and digital PCR (dPCR) using the array-based |Q| assay simultaneously.

### Clinical adjudication of bacterial coinfections.

Blood for culturing was collected from 58/161 patients suspected of an infection. Blood culture results and lab results were compared against the IMX-BVN-3 bacterial likelihood scores. A thorough chart review was performed on patients with discordant IMX-BVN-3 bacterial likelihood scores and bacterial culture results and other laboratory results to identify any patient with suspected bacterial infection. Bacterial infection was confirmed if the patient had (i) ED/inpatient primary or relevant discharge diagnoses that included any bacterial infections with use of antibiotics or sepsis or septic shock from suspected bacterial infection, (ii) positive microbiological data for bacterial pathogens collected within 48 h from ED presentation, or (iii) infectious disease expert consultation documenting bacterial infection upon hospital admission.

### Statistical analysis.

We calculated the Pearson correlation between the IMX-BVN-3/IMX-SEV-3 viral likelihood scores and severity with the absolute viral load in the nasopharynx and plasma for 89 patients described elsewhere (28), between the bacterial likelihood scores and levels of C-reactive protein, procalcitonin, and lactate dehydrogenase, and the Spearman rank correlation between the cycle threshold (*C_T_*) and the viral likelihood scores. We compared the viral loads between the true-positive and false-negative calls of viral infection as well as the severity scores between clinical outcomes using the Wilcoxon rank sum test with continuity correction and adjusted the *P* value when comparing multiple outcomes using the Benjamin and Hochberg correction. We also calculated the sensitivity, specificity, and the likelihood ratios of the viral and bacterial classification bands against the PCR COVID-19 positivity and adjudicated bacterial coinfections, respectively. Additionally, we also compared the proportions of patients in the severity likelihood bands and their clinical outcomes—disposition from the ED and the need for ventilation/30-day mortality using χ^2^ tests with continuity corrections. All analyses were performed in R.

### Ethics approval and consent to participate.

The Institutional Review Board approved protocols 55650 (Stanford ED biorepository for suspected COVID-19 patients; approved 30 March 2020) and 55924 (HostDx-ViralSeverity—a COVID-19 prognostic tool; approved 10 June 2020), and informed consent was obtained from all research participants as per regulations. All procedures were followed in accordance with the ethical standards of the responsible committee on human experimentation at Stanford University and with the Helsinki Declaration of 1975. Consent for publication was not applicable.

### Data availability.

The data sets used and/or analyzed during the current study are available from the corresponding author on reasonable request.
