# Supplementary material for: Using a 29-mRNA Host Response Classifier To Detect Bacterial Coinfections and Predict Outcomes in COVID-19 Patients Presenting to the Emergency Department
Source: Microbiol Spectr. 2022 Oct 17;10(6):e02305-22. doi: 10.1128/spectrum.02305-22 (PMC9769905; doi:10.1128/spectrum.02305-22)
Supplement: Supplemental file 1 — Supplemental material. Download spectrum.02305-22-s0001.pdf, PDF file, 0.2 MB [file spectrum.02305-22-s0001.pdf]

Supplemental Table 1. Clinical adjudication based on expert chart review in 6 patients with Possible IMX-BVN-3 bacterial scores

ID	IMX-BVN-3		IMX-SEV-3 severity result	Clinical characteristics	Microbiology findings	Antimicrobial therapy and other data	Discharge diagnoses	Bacterial infection
	Bacterial	Viral						
0076	Possible	Very Unlikely	Moderate	Septic shock on admission	Positive (*C. difficile* toxin in stool)	Cefepime, vancomycin, fidaxomycin, metronidazole	Septic shock; *C. difficile* colitis	Co-infection
0082	Possible	Possible	Moderate	Hypoxic respiratory failure, hypotension	Negative	Cefepime azithromycin, vancomycin	Septic shock; bacterial pneumonia, viral pneumonia	Co-infection<br><br>(bacterial pneumonia diagnosed clinically)
0281	Possible	Unlikely	Moderate	Abdominal pain	Positive (Urine culture positive for viridans streptococci)	Ertapenem, cefepime, metronidazole	COVID-19; abdominopelvic abscess	Co-infection<br><br>(gastrointestinal perforation with peritonitis and fecal pathogens in urine)
0397	Possible	Possible	Moderate	Hypoxic respiratory failure, persistent leukocytosis	Negative	Azithromycin, ceftriaxone	Persistent leukocytosis	Co-infection<br><br>(bacterial infection, improved with antibiotics suspected by ID consult)
0477	Possible	Possible	Moderate	Shortness of breath, hypoxia, SIRS	Negative	Eefepime, caspofungin	Sepsis; Leukemia with graft vs. host disease	Co-infection<br><br>(bacterial infection, treated with antibiotics suspected by ID consult)
0500	Possible	Very Unlikely	Moderate	Fall	Negative	None used on admission	Asymptomatic COVID-19 infection	Negative

		Blood culture, clinical microbiology data	
		Coinfection	No coinfection
IMX-BVN-3 bacterial likelihood	Very Likely/Possible	**5**	**1**
	Unlikely/Very unlikely	0	155 (**52**)

Supplemental Figure 1. Confusion matrix for the likelihood of bacterial infection predicted by IMX-BVN-3 compared to clinical findings for a subset of patients with complete microbiological data (total 58 in bold). Chart review and clinical adjudication confirmed bacterial co-infection in 5/6 patients in the Possible predictions.
